# An exploratory case study of environmental factors related to military alcohol misuse

**DOI:** 10.1186/s12889-018-5843-5

**Published:** 2018-07-20

**Authors:** Susan I. Woodruff, Suzanne L. Hurtado, Cynthia M. Simon-Arndt, Jessica Lawrenz

**Affiliations:** 1 School of Social Work, Center for Alcohol and Drug Studies and Services, San Diego State University, 5500 Campanile Drive, HH 203, San Diego, CA 92182 USA; 2 Naval Health Research Center, San Diego, CA USA; 3 Leidos, San Diego, CA USA; 4 San Diego State University Research Foundation, San Diego, CA USA

**Keywords:** Alcohol misuse, Environmental strategies, Risk factors, Protective factors, Marine Corps, Military, Alcohol accessibility, Alternative activities, Alcohol sales, Community factors

## Abstract

**Background:**

Alcohol misuse has been an ongoing issue for the US Armed Services, with the Marine Corps maintaining the highest levels of problematic drinking. Broad environmental, social, and policy factors play an important role in alcohol misuse but are rarely studied as objective measures.

**Methods:**

This case study used a pattern-matching approach to examine the associations between objective on- and off-base community environmental risk and protective factors and 4 objective alcohol-related outcomes at 3 large Marine Corps installations. The study utilized existing aggregated data from Marine Corps electronic data sources and information from internet searches of installation and community services and characteristics. Installation-level alcohol misuse outcomes included the rates of personnel receiving non-medical alcohol services, combined inpatient and outpatient alcohol-related primary diagnoses, alcohol-related domestic violence, and driving under the influence arrests. Installation-level environmental correlates included dollars spent on alcohol sales, density of alcohol outlets, extent of alternative activities, and installation and off-base sociodemographic factors.

**Results:**

In general, younger age, enlisted pay grade, and being stationed overseas were related with higher rates of alcohol-related problems among Marines. Greater on-base alcohol sales (both in bars and stores), as well as a greater density of restaurants and bars that serve alcohol, were associated with alcohol misuse outcomes. Several community factors were also associated with alcohol misuse. The hypothesized protective effects of alternative activities were inconsistent.

**Conclusions:**

Findings suggest that environmentally-oriented strategies, particularly restricting on-base sales of alcohol, may help to reduce alcohol-related harm in the Marine Corps.

## Background

Excessive alcohol use is associated with a broad range of negative health and social consequences, including productivity loss, legal problems, arrests for driving under the influence (DUI), incarceration, injury and hospitalization, motor vehicle crashes, physical aggression, domestic violence, risky sexual behavior, suicide, addiction, and death [1–5]. Alcohol misuse is a primary concern for the Department of Defense (DoD) because rates are higher among military personnel than civilians, the economic cost to the DoD is considerable, and alcohol misuse can adversely affect job performance and readiness [6]. High-risk alcohol use has been an ongoing issue for the US military, with the Marine Corps maintaining the highest levels of problematic drinking among the services [7].

Social ecological frameworks applied to health emphasize the role of not only individual-level variables but also broader environmental, social, and policy factors [8]. Specific to alcohol use, for example, the World Health Organization has recognized the importance of restricting alcohol availability and pricing strategies to reduce alcohol-related harm [9]. Social disorganization theory would predict that broad community disadvantage (e.g., higher crime, greater unemployment, and poor community infrastructure and resources) may promote higher alcohol norms and heavy drinking [10]. Understanding environmental factors related to alcohol use is important, in that combining individual-level change in knowledge, attitudes, and behaviors with change in the environment, such as availability of alcohol, laws and policies, and community norms, is considered among the strongest of prevention strategies.

Self-report survey studies have examined environmental influences and strategies for reducing alcohol risks in the military to some extent [11–13]. According to a 2012 study by the Institute of Medicine, military-relevant environmental risk factors, such as ready availability of alcohol on or near bases (often at reduced prices) and boredom on military bases and in deployed settings with few recreational activities available or perceived to be available, may also play a role [14]. In addition, cultural and geographical influences may contribute to alcohol misuse for those deployed in overseas countries [15, 16]. A study of unit-level influences on alcohol and tobacco use found that being stationed overseas, greater reported access to alcohol in barracks, reports of lower enforcement of alcohol control policies in barracks, and perceptions that drinking was the only recreation available were all factors associated with increased alcohol use among military personnel [17].

The aforementioned studies, while important, were limited in that they generally relied on service members’ subjective reports of alcohol misuse and environmental risks. Moreover, they included a limited number of alcohol outcomes and did not examine environmental protective factors. The purpose of the present study was to add to what is known about environmental correlates of alcohol misuse in the US Marine Corps by identifying a large number of objective *on-base* and *surrounding community* environmental risk and protective factors that may be associated with several alcohol misuse outcomes and amenable to change. Using an exploratory case study approach, we studied 3 large Marine Corps installations whereby objective base-level aggregated data were collected for 4 alcohol-related outcomes, a wide range of on-base and community environmental risk factors, and several environmental factors thought to potentially protect against or offset alcohol misuse among Marines.

## Methods

### Pattern-matching case study approach

Pattern matching is a qualitative case study methodology useful for the intensive study of a single case or a small number of cases (i.e., 3 Marine Corps installations in this study) [18]. Using this hypothesis-driven analytical technique, we compared an empirically-based pattern with an expected or hypothesized pattern for multiple outcomes and factors. For example, we hypothesized that a higher rank order of alcohol-related medical diagnoses would correspond to a higher rank order of the risk factor of on-base alcohol availability among the 3 installations. We examined 4 alcohol-related outcome variables and a wide variety of 23 objective environmental factors, which included 13 on-base and 10 off-base sociodemographic and environmental risk and protective factors (Table 1).

**Table 1:** Risk and protective on- and off-base correlates, by installation

Correlate	Rate per 1000, area density, or %		
	Installation		
	A	B	C
*On-base*			
Sociodemographic factors			
Region	CONUS	CONUS	OCONUS
Mean (SD) age (y)	24.9 (6.4)	25.6 (7.1)	25.9 (7.1)
% junior enlisted	37.1	35.6	27.0
% single	49	52	60
Environmental risk factors			
$ spent on open sales (per capita)	15.12	8.69	147.48
$ spent on closed sales (per capita)	327.73	309.34	558.22
Density of stores selling alcohol (per sq. mi)	0.130	0.081	0.189
Density of bars/restaurants serving alcohol (per sq. mi)	0.070	0.033	0.178
Density of alternative activities with alcohol (per sq. mi)	0.010	0.008	0.078
Environmental protective factors			
Density of all alternative activities (per sq. mi)	0.150	0.154	0.567
Density of alcohol-free alternative activities (per sq. mi)	0.140	0.146	0.489
Density of schools, parks, shops, churches (per sq. mi)	0.075	0.060	0.323
Driving restrictions for lowest ranks	No	No	Yes
*Off-base*			
Sociodemographic factors			
Median household income (USD)	62,958	38,315	20,455
Unemployment rate (%)	10.1	8.9	7.1
Total crime rate (per 1000 population)	26.8	31.1	8.8
Environmental risk factors			
Density of stores selling alcohol (per sq. mi)	2.390	1.330	0.779
Density of bars/restaurants serving alcohol (per sq. mi)	4.93	1.75	4.39
Density of alternative activities with alcohol (per sq. mi)	0.166	0.045	0.151
Environmental protective factors			
Density of alternative activities (per sq. mi)	1.300	0.517	0.362
Density of alcohol-free alternative activities (per sq. mi)	1.140	0.472	0.211
Density of schools, parks, shops, churches (per sq. mi)	4.650	2.560	0.829
Neighborhood walkability score	89	28	72

Abbreviations*: CONUS* Continental United States, *OCONUS* Outside the continental United States, *USD* US dollar

Since the pattern-matching approach relies on expected patterns, we proposed several directional hypotheses based on published studies of individual-level risk factors in civilian and military samples, as well as social ecological theory. We hypothesized the following environmental risk factors would be related to higher alcohol misuse:

- Being stationed in an overseas region [17];
- Sociodemographic characteristics, including young age, single status, and lower pay grade of Marines [19];
- Greater alcohol availability on and off base (e.g., density of stores and restaurants/bars that sell or serve alcohol) [20];
- Higher alcohol sales on base [21];
- More activities on and off base where alcohol is available [22]; and
- Other off-base surrounding community characteristics that generally indicate broad community disadvantage, including lower community wealth and resources, higher unemployment, and higher crime [23–26].

We also hypothesized the following protective factors (i.e., factors expected to offset alcohol misuse) would be associated with decreased alcohol misuse:

- Greater number of on- and off-base alternative activities, particularly those that are alcohol-free [27];
- Greater number of on- and off-base resources and infrastructure, including schools, parks, shopping centers, and churches [28–30];
- Positive community structures, such as high community walkability [31]; and
- Driving restrictions on young Marines [32].

### Sources of data and measures

In the present study, all data were already existing and either aggregated or available for aggregating. Some of the data were in raw count format and collected from various program offices at the 3 installations. Additional sources of data included existing Marine Corps electronic data sources, such as the Alcohol and Drug Management Information Tracking System and the Consolidated Law Enforcement Operations Center databases. Finally, other data were drawn from internet searches of installation and community services and characteristics. We identified existing risk/protective factors and alcohol outcomes for the 2011 calendar year for which data were available for all 3 installations. We attempted to obtain parallel information for the installation and off-base community closest to the installation. To control for differences in the installation and community population size, raw counts were converted to rates per capita or area density measures.

### Alcohol-related outcome measures

Counts of 4 alcohol-related outcome variables for 2011 were gathered for each installation. We used installation population figures from the 2011 Defense Medical Epidemiology Database [33] to compute rates of alcohol-related problems per 1000 Marines. Alcohol misuse outcome variables in 4 sectors included:

- One clinical outcome: rate of Marine Corps personnel receiving non-medical alcohol services, including alcohol education, outpatient services, or intensive outpatient services as reported by the installation’s Substance Abuse Counseling Center or the Substance Abuse Rehabilitation Program;
- One medical outcome: rate of combined inpatient and outpatient alcohol-related primary diagnoses reported for Marines in the Career History Archival Medical and Personnel System database [34];
- One social/interpersonal outcome: rate of alcohol-related domestic violence for Marines reported by Headquarters, Marine Corps (HQMC); and
- One legal outcome: rate of DUI arrests for Marines based on data from the installation’s military police database.

### On-Base sociodemographic and environmental risk factors

Sociodemographic variables at the on-base level included: (a) region where the installation is located, defined as the continental United States (CONUS) or outside the continental United States (OCONUS); (b) mean age of Marines at the installation, based on the 2008 DoD Survey of Health Related Behaviors Among Active Duty Military Personnel [35]; (c) percentage of junior enlisted personnel (E1–E3 and E4–E6), based on the 2008 DoD Health Related Behaviors Survey [35]; and (d) percentage of personnel who were single, as reported by HQMC [36]. Gender was not included as a risk/protective factor because of the low percentage of female Marines.

On-base environmental risk factors for alcohol misuse outcomes included the following: (a) dollar amount spent on alcohol in bars/restaurants (i.e., open sales) per capita, according to data provided by HQMC and Marine Corps Community Services (MCCS) on each installation; (b) dollar amount spent on alcohol sales in stores and outlets (i.e., closed sales) per capita, reported by HQMC and MCCS on each installation; (c) density of stores selling closed alcohol per square mile based on MCCS web pages and/or publications for each installation; (d) density of restaurants/bars serving alcohol per square mile based on comprehensive lists of on-base restaurants/bars; and (e) density of alternative activities where alcohol is served per square mile based on comprehensive lists of on-base services and activities, such as bowling alleys and other recreational activities.

### On-Base environmental protective factors

On-base protective factors included: (a) density of all alternative activities per square mile based on comprehensive lists of on-base services and activities provided by MCCS; (b) density of alcohol-free alternative activities per square mile based on comprehensive lists of on-base services and activities from MCCS; (c) density of the combined number of schools, parks/trails, shopping centers, and churches per square mile based on MCCS and other base publications; and (d) presence of driving restrictions for lower-ranking enlisted personnel.

### Off-Base sociodemographic and environmental risk factors

Sociodemographic characteristics of the communities surrounding the installations included the following based on internet sources: median household income, unemployment rate, and density of alternative activities where alcohol is served per square mile; total crime rate based on official online crime statistics; density of stores selling closed alcohol per square mile based on state and Marine Corps publications; and density of restaurants/bars serving alcohol per square mile based on internet sources and reviews of online menus. Community alcohol sales could not be ascertained and, therefore, were not included as a risk factor.

### Off-Base environmental protective factors

Off-base community environmental protective factors were determined based on internet sources and community and installation publications. These measures consisted of: (a) density of all alternative activities per square mile; (b) density of alcohol-free alternative activities per square mile; (c) density of the combined number of schools, parks/trails, shopping centers, and churches per square mile; and (d) a neighborhood walkability score from walkscore.com. This website uses an algorithm that weighs the distance of amenities and other common destinations within a 30-min walking distance from the community’s zip code.

### Analysis

We quantified all measures and ranked each from low to high by installation. We then applied pattern matching to compare rank ordering of the observed environmental correlate and the alcohol outcome for the 3 installations. If the rank ordering of the correlate and outcome matched in the expected hypothesized direction, it was counted as a match. Directions of matched associations could be positive or negative (inverse). When the empirically-based and expected pattern did not match, we did not consider the finding to be a meaningful association.

## Results

The 3 installations varied in their rates of clinical, medical, social/interpersonal, and legal alcohol-related outcomes. The clinical outcome rate of personnel receiving educational and outpatient alcohol services ranged from 27.4–48.1 per 1000 Marines across the 3 installations. The medical outcome (rate of alcohol-related inpatient and outpatient primary diagnoses) ranged from 28.3–40.8 per 1000 Marines. The rate of alcohol-related domestic violence, the social/interpersonal outcome variable, ranged from 0.8–2.9 per 1000 Marines. The DUI rate (legal outcome) ranged from 1.44–12.5 per 1000 Marines.

Table 1 presents sociodemographic and aggregated environmental risk and protective factors at the installation and community level that either theory or individual-level self-report studies suggest could potentially influence alcohol outcomes. Installations A and B were CONUS; installation C was OCONUS. Installation C had a lower proportion of junior enlisted pay grade personnel and a higher percentage of single Marines than installations A and B. With regard to on-base environmental risk factors, installations A and B had lower alcohol sales (both open and closed) and accessibility (densities of outlets selling and serving), while installation C had higher rates of these types of risk factors. However, installation C also had the highest density of on-base environmental protective factors, such as alternative activities (including alcohol-free activities), a higher density of on-base resources, such as parks and schools, and driving restrictions on personnel in lower ranks.

Off-base communities surrounding the installations varied in sociodemographic characteristics, with Installation A having a greater household income on average, a higher unemployment rate, a moderate crime rate, and a higher density of stores and bars serving alcohol. Installation A was also highest for several protective factors, such as the availability of alcohol-free alternative activities, density of community resources, such as schools, parks, and shops, and a high walkability score.

Table 2 presents pattern-matched associations between rates of the 4 alcohol outcome measures and on- and off-base environmental correlates. The first column gives expected associations as either positive (+) or inverse (−) associations. In the observed pattern columns, associations that aligned with the expected pattern are circled. Associations that could not be interpreted as either positive or inverse were considered neutral (+−). The clinical outcome was associated in the expected direction with 8 correlates, the medical outcome was associated with 6 correlates, the interpersonal outcome was associated with 4 correlates, and the legal outcome was associated with 3 potential correlates.

**Table 2:** Results of pattern matching between expected and observed environmental correlates with alcohol-related outcomes

Environmental correlate	Pattern				
	Expected	Observed			
		Clinical outcome	Medical outcome	Interpersonal outcome	Legal outcome
*On-base*					
Sociodemographic factors					
Region (OCONUS)	+	⊕	⊕	+−	+−
Age	–	⊖	+−	⊖	+−
% junior enlisted	+	⊕	–	⊕	+−
% single	+	⊕	⊕	–	+−
Environmental risk factors					
$ spent on open sales	+	⊕	⊕	+−	+−
$ spent on closed sales	+	⊕	⊕	+−	+−
Density of stores selling alcohol	+	+−	+−	+−	+−
Density of bars/restaurants serving	+	⊕	+−	+−	+−
Density of alternative activities with alcohol	+	+−	+−	+−	+−
Environmental protective factors					
Density of all alternative activities	–	+	+	+−	+−
Density of alcohol-free activities	–	+−	+−	+−	+−
Density of schools, parks, etc.	–	+	+	+−	+−
Driving restrictions for low ranks	–	+−	+−	+−	⊖
*Off-base*					
Sociodemographic factors					
Household income	–	+−	⊖	+	+
Unemployment rate	+	–	+−	⊕	+−
Crime rate	+	–	–	+−	+−
Environmental risk factors					
Density of stores selling alcohol	+	+−	+−	⊕	+−
Density of bars/restaurants serving	+	+−	+−	+−	⊕
Density of alternative activities with alcohol	+	+−	+−	+−	⊕
Environmental protective factors					
Density of all alternative activities	–	+−	+−	+	+−
Density of alcohol-free activities	–	+−	+−	+−	+−
Density of schools, parks, etc.	–	⊖	⊖	+	+−
Neighborhood walkability	–	+−	+−	+−	+

Abbreviations*: OCONUS* Outside the continental United States

*+* positive association, *+ −* neutral/no association, *−* inverse association

Higher rates of personnel receiving alcohol-related clinical services were associated with OCONUS location and having a larger proportion of young, junior enlisted, and single personnel, as expected. In addition, more money spent on alcohol in on-base bars and from on-base stores was associated with a higher rate of use of clinical services. A higher rate of alcohol-related clinical services was also associated with on-base accessibility of alcohol, as measured by greater density of bars and restaurants serving alcohol. Greater density of on-base alternative activities (with and without alcohol), as well as greater density of on-base infrastructure, was unexpectedly associated with higher rates of alcohol-related clinical services. In addition, several off-base sociodemographic factors were unexpectedly associated with higher rates of Marines’ use of alcohol clinical services (e.g., lower community crime and unemployment). Community environmental risk and protective factors were not associated with the rate of use of alcohol-related clinical services, with the exception of community infrastructure and resources; a relatively higher density of schools, parks, shopping, and churches in the community were associated with lower alcohol clinical utilization, as hypothesized.

Table 2 presents results for the medical outcomes of alcohol-related inpatient and outpatient diagnoses for Marines. Four on-base factors were associated with higher rates of alcohol-related diagnoses: OCONUS status, more single personnel, and greater amounts of money spent on alcohol at on-base bars/restaurants and on closed sales of alcohol. Lower community wealth and resources, in the form of schools, parks, shopping, and churches, were inversely associated with higher alcohol-related medical diagnoses, although most community factors were unrelated. There was an unexpected association of a greater density of on-base alternative activities (with and without alcohol), as well as greater density of on-base infrastructure, associated with higher rates of alcohol-related inpatient and outpatient diagnoses.

With regard to interpersonal outcomes (alcohol-related domestic violence), 4 of the on- and off-base correlates were associated in the expected direction (Table 2). Younger and junior enlisted personnel, a higher community unemployment rate, and a higher community density of stores selling alcohol were all associated with higher rates of alcohol-related domestic violence. All on-base environmental risk and protective factors were not related to the rate of alcohol-related domestic violence, and the community environmental protective factors of the density of alternative activities and community resources were associated in an unexpected direction.

DUI rate, the legal outcome in this study, was associated in an expected direction with 3 correlates (Table 2). DUI rates were: lower where driving restrictions for young, enlisted personnel were in place; higher where there was a greater density of off-base community bars and restaurants serving alcohol; and higher where there was a greater density of community alternative activities with alcohol present. Most of the on- and off-base environmental correlates were not associated with DUI rates.

## Discussion

In the present study, there was variability in the number of risk and protective environmental factors associated in the hypothesized direction with the 4 alcohol-related outcomes, ranging from 8 correlates for the clinical outcomes to 3 for legal outcomes, out of a possible 23 correlates examined. Marines’ sociodemographic factors, on-base open and closed sales of alcohol, and off-base infrastructure (i.e., density of schools, parks, etc.) emerged as the most consistent correlates, with each of these factors being associated with 2 of the 4 alcohol-related outcomes.

Marines stationed overseas were at a greater risk than those stationed in the United States for receiving alcohol-related clinical services and diagnoses, a consistent finding with previous alcohol-misuse prevalence studies based on self-reported behavior [7, 17]. Several other sociodemographic factors found to be related to alcohol misuse in the present study were generally in line with our hypotheses, as well as results from other studies conducted at the individual level [7, 19]. A greater proportion of young Marines was associated with higher rates of alcohol-related clinical services and interpersonal outcomes (i.e., domestic violence). Having more single Marines on base was associated with a greater usage of alcohol clinical services and medical diagnoses. These findings provide support for the continuation of selective prevention and risk-reduction efforts for Marines in these demographic subgroups.

Community sociodemographic characteristics did not result in expected matched patterns with Marines’ alcohol outcomes with 2 exceptions: lower community wealth with higher medical outcomes and higher unemployment rates with higher interpersonal outcomes. It may be unreasonable to expect community sociodemographic and economic factors to be strongly associated with alcohol outcomes, given that Marines, particularly those who are younger and most at risk, may be separated from their surrounding communities in physical, cultural, and social ways (especially if overseas).

Consistent with our hypothesis, the data showed that greater on-base alcohol sales (both open and closed) were associated with higher rates of alcohol-related clinical services and medical diagnoses; however, there was no association, or a lack of pattern matching, with rates of alcohol-related domestic violence and DUI. Although a causal relationship cannot be assumed, these matched positive associations suggest that reducing the amount of money spent on alcohol on base could be related to a concomitant reduction in clinical and medical alcohol outcomes.

Availability and accessibility of alcohol are well-documented environmental risk factors for alcohol misuse in civilian studies [20, 37, 38] and, therefore, were expected to be associated with alcohol-related outcomes. A greater density of on-base restaurants/bars serving alcohol was associated with higher rates of Marines receiving alcohol-related educational/outpatient clinical services. At the community level, a greater density of off-base restaurants and bars selling alcohol was associated with higher alcohol domestic violence, and a greater density of off-base restaurants and bars serving alcohol was associated with higher DUI rates. Installation driving restrictions on young personnel appeared to be protective in that it was specifically related to lower DUI rates among Marines.

A common environmental strategy for reducing alcohol problems is to increase the number of alcohol-free alternative activities that people can participate in, thereby reducing the opportunity to drink during that time. Conversely, more activities that involve alcohol may contribute to alcohol problems. This concept was supported by our finding that more off-base activities involving alcohol were associated with higher DUI rates among Marines, although a similar pattern was not seen for on-base activities. In fact, on-base alternative activities with alcohol were not related to any of the alcohol outcomes, and all on-base alternative activities (with and without alcohol) were associated in the direction not aligned with our hypotheses.

Infrastructures, such as parks and shopping areas, were hypothesized to be protective against alcohol problems, although this relationship was only found at the community level for clinical and medical outcomes and not at the on-base level. It is likely that other factors served as stronger influences on drinking and alcohol-related behaviors than did on-base infrastructures, thus reducing any meaningful impact of infrastructures on behavior. In addition, peer norms among Marines that do not emphasize utilization of on-base resources may reduce the likelihood of their use and impact.

The most notable strength of the study was its consideration of a wide variety of objective environmental factors and 4 objective indicators of installation-level alcohol problems. The differential association of the correlates by the 4 outcomes suggests that these alcohol outcomes may be distinct, each with its own risk and protective influences. Our use of base-level data from both official, centralized sources and those reported at the local installation level is a major strength and has not often been applied in research on alcohol misuse in the military. An additional strength of the study was the measures were unobtrusive and nonreactive since no direct data collection from individual Marines was required; rather, existing secondary data sources were used.

At the same time, studies using aggregated data cannot provide information on individual motivations or thought processes regarding drinking behavior, nor do they allow for separating individual-level and environmental contributions to alcohol use outcomes. This study had some additional limitations. Most measures were collected for the 2011 timeframe, although several installation-level demographic variables were only available for the earlier time period of 2008. The quality and completeness of alcohol misuse incident data that were collected or reported may vary by installation. Local or regional statutes and norms, as well as informal military and civilian law enforcement arrangements, may affect how alcohol-related incidents are handled and reported, and underreporting may have occurred. Variation by installation and data sources could affect the alcohol-related misuse rates, as well as the observed associations with environmental factors and the validity of our findings.

The small number of installations examined in this study restricted the types of analyses that could be performed using the aggregated data. For this reason, we used a pattern-matching approach suitable for an exploratory case study that might uncover interpretable patterns. The validity of the pattern matching case study approach relies on assessing the correspondence between a theoretical pattern with the observed pattern. While we included environmental factors that have strong evidence of association with alcohol misuse from other empirical studies and limited theory, we do not know of a comprehensive explicit model of the phenomenon and may have not included all important influences. It is noteworthy that the 3 installations in the study are among only 10 Marine Corps bases that had a base population of at least 5000 active duty personnel. Even so, replication with a larger sample is essential to determine whether these findings are robust. A final limitation is that the cross-sectional nature of the study offers little information about cause and effect. However, establishing associations between environmental risk and protective factors and adverse alcohol-related outcomes lays the foundation for subsequent work to establish these causal relationships.

The results from the present study suggest recommendations for enhanced environmental interventions that are in the purview of the Marine Corps and in line with recent restrictions on the availability and accessibility of alcohol on Marine Corps bases [39]. Although sociodemographic characteristics are not typically thought of as environmental factors, they are important to consider when identifying high-risk groups. Because we found that several sociodemographic factors, including age and enlisted pay grade, were related to alcohol-related outcomes, continuing to target selective prevention and risk-reduction efforts at Marines in these demographic subgroups is warranted. We recommend continued expansion of alternative structured activity options, as well as selective prevention and educational programs that target young Marines in junior pay grades.

Higher on-base alcohol sales were associated with alcohol-related clinical outcomes and medical diagnoses, which suggests that a decrease in the sale of alcohol on base, by reducing the density of outlets, increasing the price of alcohol, and/or reducing the operation hours of outlets, may decrease alcohol misuse. However, it is unknown how restrictions of this type might affect off-base alcohol purchasing and alcohol-related incidents, which highlights the importance of conducting pilot studies that examine the effects of these environmental interventions.

## Conclusions

These findings suggest that environmentally-oriented strategies, particularly restricting on-base alcohol sales, may help to reduce alcohol-related harm in the Marine Corps. More on- than off-base factors were related to alcohol outcomes; yet, even community factors may play a role in risk and mitigating risk. This study provides empirical evidence that further supports and strengthens ongoing environmental efforts for reducing alcohol problems among Marines.

## Abbreviations

CONUS: Continental United States
DoD: Department of Defense
DUI: Driving under the influence
HQMC: Headquarters, Marine Corps
MCCS: Marine Corps Community Services
OCONUS: Outside the continental United States
